# Slow but not low: genomic comparisons reveal slower evolutionary rate and higher dN/dS in conifers compared to angiosperms

**DOI:** 10.1186/1471-2148-12-8

**Published:** 2012-01-20

**Authors:** Emmanuel Buschiazzo, Carol Ritland, Jörg Bohlmann, Kermit Ritland

**Affiliations:** 1 Department of Forest Sciences, University of British Columbia, 2424 Main Mall, Vancouver, BC V6T 1Z4, Canada; 2 School of Natural Sciences, University of California, Merced, 5200 North Lake Road, Merced, CA 95343 USA; 3 Michael Smith Laboratories, University of British Columbia, 2185 East Mall, BC V6T 1Z4, Canada

## Abstract

**Background**

Comparative genomics can inform us about the processes of mutation and selection across diverse taxa. Among seed plants, gymnosperms have been lacking in genomic comparisons. Recent EST and full-length cDNA collections for two conifers, Sitka spruce (*Picea sitchensis*) and loblolly pine (*Pinus taeda*), together with full genome sequences for two angiosperms, *Arabidopsis thaliana *and poplar (*Populus trichocarpa*), offer an opportunity to infer the evolutionary processes underlying thousands of orthologous protein-coding genes in gymnosperms compared with an angiosperm orthologue set.

**Results**

Based upon pairwise comparisons of 3,723 spruce and pine orthologues, we found an average synonymous genetic distance (dS) of 0.191, and an average dN/dS ratio of 0.314. Using a fossil-established divergence time of 140 million years between spruce and pine, we extrapolated a nucleotide substitution rate of 0.68 × 10^-9 ^synonymous substitutions per site per year. When compared to angiosperms, this indicates a dramatically slower rate of nucleotide substitution rates in conifers: on average 15-fold. Coincidentally, we found a three-fold higher dN/dS for the spruce-pine lineage compared to the poplar-*Arabidopsis *lineage. This joint occurrence of a slower evolutionary rate in conifers with higher dN/dS, and possibly positive selection, showcases the uniqueness of conifer genome evolution.

**Conclusions**

Our results are in line with documented reduced nucleotide diversity, conservative genome evolution and low rates of diversification in conifers on the one hand and numerous examples of local adaptation in conifers on the other hand. We propose that reduced levels of nucleotide mutation in large and long-lived conifer trees, coupled with large effective population size, were the main factors leading to slow substitution rates but retention of beneficial mutations.

## Background

Determining the mutational and the selective forces responsible for evolution has overarching implications in biology, e.g. in understanding what makes species unique and how organisms respond to biotic and abiotic challenges. Identifying the rate of evolution and the patterns of nucleotide substitution underlying DNA evolution has thus become a fundamental goal of molecular genomics [1,2]. Key to the central dogma of molecular biology, protein-coding sequences (hereafter referred to as genes) have classically been regarded as a major unit of evolution. Substitutions at synonymous (silent) and non-synonymous (replacement) sites are commonly distinguished to differentiate between neutral (or at least weak) and active selective forces acting on genes, respectively. In pairwise comparisons of orthologous genes, the ratio of non-synonymous distance (i.e. number of substitutions per non-synonymous site; dN) over synonymous distance (dS) gives a general but conservative indication of the mode and strength of selection [1,2]. An excess of non-synonymous substitutions (dN/dS > 1) suggests adaptive or diversifying selection, while an excess of synonymous mutations (dN/dS < 1) indicates purifying selection, and no difference between synonymous and non-synonymous mutation rates (dN/dS = 1) is taken as evidence for neutrality [3].

Large-scale sequence datasets now exist, allowing comparisons to be made for thousands of genes in all domains of life. Synonymous and non-synonymous substitution rates have been found to vary widely within and between taxa [4-7]. From early studies based on a limited number of species and genes to the era of genomics and systems biology [8,9], a complex blend of non-mutually exclusive biological, biochemical and demographic mechanisms emerged to explain these variations. While intraspecies differences are believed to be influenced by selection on protein structure and function (reviewed in [10-14]), interspecies differences are influenced by (i) the efficacy of the DNA repair machinery, (ii) life history traits (e.g. generation time), (iii) metabolic rate, (iv) effective population size (random genetic drift), (v) purifying (background) selection and (vi) reproductive strategy. Some factors (i - iii) influence the way mutations appear, while others (iv - vi) influence their fixation over generations (reviewed in [9,13,14]).

Among plants, most of the attention in comparative evolutionary studies has been focused on flowering plants [4,5,14,15], and interest is now growing for other plant taxa as more sequence data is produced. Gymnosperms are separated from angiosperms by ~300 million years of evolution [16]. Expectedly, many biological features of gymnosperms and angiosperms differ greatly, including seed morphology, life span, diversification rate, pollination processes, environmental requirements and response to environmental stresses. With ~600 extant species, conifers make up about two thirds of all gymnosperm species, and are the dominant plants in most temperate and boreal ecosystems. Conifers have an immense ecological and economical value such as practical forestry economics, immediate ecological value of forest ecosystems and in the long term, large capacity for carbon sequestration. Biological differences between angiosperms and conifers and the need for long-lived conifer species to cope with challenges such as insect pests and environmental changes, underscore the importance of understanding the molecular and functional evolution of conifer genomes.

The genetic architecture of conifers has been addressed by a wide variety of studies, mainly in pine (*Pinus *[17]) and spruce (*Picea *[18]). Approaches include quantitative trait locus mapping [19-21], candidate gene approaches [22,23], association mapping [24,25], BAC sequencing [26,27], transcriptome analysis [28,29], characterization of gene families [30] and proteome analyses [31], and combinations thereof [32]. Missing from past endeavors, however, are large-scale comparative comparisons that investigate both evolutionary rates and the selective forces acting on conifer genes.

In this study, we take advantage of the existing large and high-quality sequence data in two conifer species, Sitka spruce (*Picea sitchensis*) and loblolly pine (*Pinus taeda*), consisting of a collection of *bona fide *full-length cDNA sequences (FL-cDNAs) [33,34] and UniGenes constructed from several EST libraries, respectively. Together with whole-genome gene sets available for two angiosperms, *Arabidopsis thaliana *and *Populus trichocarpa*; a rich data set exists to identify rates and patterns of evolution between conifer species and between conifer and angiosperm species. We find evidence for significantly slower evolutionary rates in conifers. In stark contrast, we find a significantly higher dN/dS ratio in conifers as compared to angiosperms, indicating perhaps higher adaptation. We also investigate these patterns across functional categories of genes.

## Methods

### Protein-coding sequences for conifers and angiosperms

#### Conifer sequences

Clustered ESTs from loblolly pine were downloaded from NCBI UniGene (build 10, which had 18,921 clusters). Sitka spruce FLcDNAs came from the Treenomix II project [35]; as of Nov. 10 2009, this collection comprised 10,665 FLcDNAs, of which 3,218 clustered in contigs. We used all individual FLcDNAs because our approach ultimately removes any redundant or duplicated sequences.

#### Open reading frame (ORF) search in conifer genes

All possible ORFs (from start to stop codons) found in spruce FLcDNAs were queried against the plant UniProtKB SwissProt and trEMBL datasets [36], with predicted proteins from Sitka spruce [33] removed from the trEMBL dataset. Only ORFs from the 5,680 spruce FLcDNAs that had no hit against the SwissProt dataset were queried against the trEMBL dataset. ORFs from 3,296 spruce FLcDNAs had no homology with either of the plant UniProtKB datasets; for those, the longest ORF was arbitrarily selected for further analysis. A single FLcDNA with no ORF structure in its sequence was discarded.

We did not use the same strategy for loblolly pine because the pine UniGene set may contain only a truncated portion of the actual coding sequence. For conifers, we looked in each member of the UniGene set for the ORF among all possible ORFs with the same frame as the longest overlapping sequence with the best-scoring BLAST query against the spruce ORFs. Of 18,921 pine UniGenes, we found 7,627 ORFs in the same frame as spruce ORFs.

#### Orthology of conifer genes

We used the reciprocal best hit (RBH) approach [37,38] to infer putative 1:1 orthologues between spruce FLcDNAs and pine UniGene sequences, using BLAST with -e threshold = 10^-20^. We found a total of 4,774 RBHs, of which 4,250 contained a complete ORF in pine.

#### Angiosperm orthologues

*A. thaliana *was chosen because it represents the best characterized plant genome. Poplar was included in the analyses as the first completely sequenced tree genome. *A. thaliana *coding sequences were downloaded from the TAIR9 annotation release [39]. Poplar coding sequences (annotation 1.1) were downloaded from the JGI Genome Portal [40]. We used Ensembl Compara predictions through the BioMart server [41] to select a list of orthologous genes from *Arabidopsis *and poplar. Only 1:1 and apparent 1:1 orthologous coding sequences were retained for analysis, finalizing a set of 5,108 orthologues.

#### Alignment

Gymnosperm (spruce-pine) and angiosperm (*Arabidopsis*-poplar) orthologous coding sequences were aligned using DIALIGN-TX [42] with highest sensitivity (-L option). Gaps in the alignments and gap-free regions > 7 bp, interpreted as non-homologous by DIALIGN-TX, were excluded from the analysis. Finally, alignments shorter than 30 amino acids were discarded. The RBH conifer orthologue set contained 3,883 alignments and the angiosperm gene set totaled 5,073 successfully aligned 1:1 orthologues.

### Data analysis

#### Substitution rates

Pairwise distances at non-synonymous (dN), synonymous (dS) and 4-fold degenerate (4D) sites (d4) were estimated for individual genes in both gymnosperm and angiosperm alignment sets using codeml (PAML 4.0) [43,44], with settings seqtype = 1, CodonFreq = 2, Runmode = -2, and transition-transversion ratio (κ) estimated from the data. Genes showing signs of saturated divergence were excluded because codeml results are reliable for moderate ranges of sequence divergence. For conifers, we discarded 42 orthologues with dN/dS = 98.99 and 118 with dS > 0.5, and for angiosperms, we discarded two genes with dN > 5 and 996 genes with dS > 4. Threshold dS values were determined by plotting dN as a function of dS and excluding outliers from the main distribution. Final RBH orthologue sets (see Additional file 1) contained 3,723 conifer genes (average gap-free length = 510 bp) and 4,080 1:1 angiosperm genes (average gap-free length = 387 bp). 95% confidence intervals for evolutionary estimates were calculated based on 1,000 bootstrap replicates using R [45]. Absolute rates of substitution at coding sites (μ) in pairwise comparisons were inferred using the formula:

$$\mu =\frac{\text{d}}{2\text{T}}$$

with d the distance at synonymous (dS), non-synonymous (dN) or 4D (d4) sites; T divergence time between spruce and pine, or between *Arabidopsis *and poplar. Divergence times are documented from fossil records, between ~120 and ~160 MYA for conifers [46-51], and between ~105 and ~115 MYA for *Arabidopsis *and poplar [52]. Unless mentioned otherwise, we used 140 MYA and 110 MYA, respectively, as working divergence times.

#### Analyses of functional categories

Functions of conifer orthologues were inferred using analogy with *Arabidopsis *proteins for GO annotations, and with plant proteins for descriptive annotation. In detail, spruce ORFs were queried against the TAIR9 protein-coding genes and the plant UniprotKB database using BLASTX (-e threshold = 10^-5^). Of the 3,983 best hits against *Arabidopsis*, 1,230 contained an ORF that successfully aligned to loblolly pine ORFs and were assigned the GO annotation corresponding to that of the best *Arabidopsis *hits, when available.

For statistical comparisons among conifer genes, we used gene set enrichment analysis tools in the Babelomics platform [53], a web application that implements threshold-independent statistics (FatiScan and logistic regression) to investigate asymmetrical distributions of GO terms, KEGG pathways and InterPro domains within our list of annotated genes ranked by dN/dS. Fatiscan uses a Fisher exact test over a collection of partitions of the ranked list of genes, while the logistic model is used to find association of each functional block with the high or low values of the ranked list; under- and over-represented functional terms are then extracted. Prior to these analyses, we removed 43 genes that showed no non-synonymous substitution. For other functional analyses, we used the 'GO Slim' classification system provided by TAIR database [54].

## Results

### Substitution rates in conifer protein-coding genes

We aligned the sequences of 3,723 spruce-pine orthologous genes and inferred the number of pairwise synonymous (dS) and non-synonymous (dN) substitutions per site (see Table 1, Additional file 1). Mean dS was 0.191 (95% confidence interval [CI] = 0.188, 0.193), meaning that on average, one mutation occurred about every five sites along both lineages since the common ancestor. Mean dN was lower than dS (0.049; CI = 0.048, 0.050), reflecting the expected elevated mutational constraint on non-synonymous sites.

**Table 1:** Substitution rates in conifer protein-coding genes compared to angiosperm genes

Pairwise comparison	Gene number	dS	d4	dN	**μ**_**S **_**(×10**^**-9**^**)**	**μ**_**4D **_**(×10-**^**9**^**)**	**μ**_**N **_**(×10**^**-9**^**)**	dN/dS
Gymnosperms:	3,723	0.1908	0.1769	0.0492	0.68	0.64	0.18	0.3137

Sitka spruce								

Loblolly pine								

Angiosperms:	4,080	2.1846	2.0057	0.2019	9.93	9.12	0.92	0.0924

*Arabidopsis*					17.02	15.63	1.57	

Poplar					2.84	2.61	0.26	

**Fold-change**								

Angiosperm:conifers		11.4:1	11.4:1	4.1:1	14.6:1	14.4:1	5.2:1	1:3.4

*Arabidopsis*:conifers					25.0:1	24.7:1	9.0:1	

Poplar:conifers					4.2:1	4.1:1	1.5:1	

Mean genetic distances at synonymous (dS), 4-fold degenerate (d4) and non-synonymous (dN) sites are expressed as a number of substitutions per site. Absolute substitution rates are expressed in substitutions per synonymous (μ_S_), four-fold degenerate (μ_4D_) and non-synonymous (μ_N _) site per year. Species-specific rates for angiosperms were estimated based on the 1:6 difference in evolutionary rate between poplar and *Arabidopsis *[57,58].

Based on fossil records, the *Pinus*-*Picea *divergence occurred between 120 and 160 MYA [46-51]. Assuming an average divergence time of 140 MYA and that rates were equivalent along both lineages, we inferred an average rate of 0.68 × 10^-9 ^(95% CI = 0.67 × 10^-9^, 0.69 × 10^-9^) substitutions per site per year at synonymous sites (μ_S_, see Table 1). However, to fully account for the uncertainty of divergence time between pine and spruce, we also consider that this time is between 120 and 160 MYA, giving the actual estimate of μ_S _as lying between 0.60 × 10^-9 ^and 0.80 × 10^-9^.

The neutral theory of molecular evolution predicts that the evolutionary rate at neutral sites corresponds to the actual mutation rate in an organism [55]. Because neutrality at synonymous sites is disputed [56], distance in a subset of synonymous sites known as 4-fold degenerate (μ_4D_) sites (i.e. sites where a change to any of the four nucleotides will not alter the amino acid during translation) stands as a better proxy to estimate the mutation rate. From our comparison in conifers, we inferred distance at μ_4D_ sites (d4) at 0.177 (95% CI = 0.174, 0.179), which translates into a substitution rate of 0.64 × 10^-9 ^per 4D site per year (μ_4_, see Table 1), and a range of 0.55 × 10^-9 ^and 0.74 × 10^-9 ^using the extreme estimates of divergence time between spruce and pine.

### dN/dS in conifer protein-coding genes

Ideally, dN/dS should be estimated at every site to find evidence of selection (which is only possible when comparing more than two species in a phylogenetic context) and not averaged over the entire gene. However, an over-representation of non-synonymous substitutions can be used as a crude indication of either adaptive evolution or at least relaxed constraint in protein-coding genes. Mean dN/dS in conifer genes was 0.314 (95% CI = 0.299, 0.329). Of the 3,723 pairwise comparisons, 100 (2.68%) had a dN/dS > 1 (Additional file 2). We note the presence of genes that are involved in abiotic and biotic stress response; some examples are protein kinases, protein phosphatases, heat shock proteins, leucine-rich repeat proteins, histone modification proteins, glycosyltransferases, and transcription factors (see Table 2).

**Table 2:** Conifer genes involved in defense, resistance and response against insects with dN/dS > 1

Spruce clone ID	Pine UniGene ID	dN/dS	UniProt ID	Species	Putative function
WS02821_B21	DT625383	7.3061	A7P5L0	*Vitis vinifera*	Protein phosphatase/Serine/threonine phosphatases

WS0297_D22	CX645632	7.0185	A7QNM9	*Vitis vinifera*	leucine-rich repeat family protein/binding protein

WS02725_C02	DR097823	6.5839	A7P656	*Vitis vinifera*	Protein phosphatase 2C/hydrolase/metal-binding

WS02757_H19	DR165429	4.4902	Q9SE11	*Funaria hygrometrica*	Chloroplast-localized small heat shock protein (HSP20) family

WS02758_N18	DR160912	4.4589	Q0DTD2	*Oryza sativa *subsp. *japonica*	Heat shock protein DnaJ

WS02741_E07	DT634060	3.3785	Q588B8	*Cryptomeria japonica*	Glycoside Hydrolase Family 17

WS02761 N01	CO365391	3.0817	A7PWA7	*Vitis vinifera*	Heat shock protein DnaJ

WS02817_M06	DR093347	2.9656	A7NWZ2	*Vitis vinifera*	serine/threonine-specific protein kinase

WS0272_J12	DR015390	2.3311	A7QFY4	*Vitis vinifera*	Heat shock protein DnaJ

WS0454_E20	DR049906	2.2728	A0MMD5	*Litchi chinensis*	Xyloglucan endotransglycosylase (Glycoside hydrolase family)

WS02774_M01	DR060506	2.1005	Q6VAA9	*Stevia rebaudiana*	UDP-glycosyltransferase

WS02749_F04	CO164226	1.7421	Q9MA24	*Arabidopsis thaliana*	Glycosyltransferase

WS0288_C08	DR022129	1.5822	A7QTB5	*Vitis vinifera*	Glycoside hydrolase

WS0292_O15	DR681862	1.294	A7P0R3	*Vitis vinifera*	heat shock protein (hsp70)

WS02729_N15	DR689530	1.1356	Q8LHS7	*Oryza sativa *subsp. *japonica*	Histone deacetylase

WS02716_E18	AI784893	1.1314	A5AWM3	*Vitis vinifera*	Pathogenesis-related transcriptional activator PTI6

WS0298_F15	DT638459	1.0692	A7QTU5	*Vitis vinifera*	Glycosyltransferase

WS02725_E03	U39301	1.0481	A0ERF9	*Cathaya argyrophylla*	Caffeic acid ortho-methyltransferase

Genes with dN/dS lower than 1 can in fact be under positive selection at specific sites [3] and dN/dS measured over the whole gene length is thus considered too conservative to identify genes or groups of genes putatively under positive selection. Hence, we also applied a segmentation test and a logistic regression test to look for functional groups of genes that are significantly and coordinately associated to high and/or low values of dN/dS. Based on 1,230 GO-annotated conifer genes, we found that heat shock proteins, genes involved in signal transduction and regulation of transcription and nucleic acids seem more likely to evolve under reduced constraint; whereas genes involved in translation, protein assembly, chlorophyll biosynthesis and cellular organization are under strong selective constraint (Additional File 3).

### Comparison between gymnosperms and angiosperms

We compared evolutionary distances between two representative conifer taxa, Sitka spruce and loblolly pine, and two representative angiosperm taxa, *Arabidopsis *and poplar (see Table 1). Mean dN in 4,080 *Arabidopsis*-poplar orthologous genes was 0.202 (95% CI = 0.199, 0.205), mean dS was 2.184 (95% CI = 2.164, 2.206), and mean d4 was 2.006 (95% CI = 1.985, 2.026). Based on a relatively confident divergence time of ~110 million years [52], we inferred an average synonymous mutation rate μ_S _of 9.93 × 10^-9 ^substitutions per year along the lineages separating *Arabidopsis *and poplar (CI = 9.84 × 10^-9^, 10.03 × 10^-9^). This is 15-fold higher than the average mutation rate found in conifer orthologues (see Table 1). Even using the lowest estimate of divergence time between spruce and pine, μ_S _is more than 10-fold higher in angiosperms. Absolute rates of substitution are calculated assuming equal rates on the poplar and the *Arabidospis *lineages, but it has been suggested that the evolutionary rate in the poplar branch is one-sixth that of the *Arabidopsis *branch since divergence [57,58]. Using this factor, we obtained μ_S _estimates of 2.84 × 10^-9 ^in the poplar lineage and 1.70 × 10^-8 ^in the *Arabidopsis *lineage (Additional file 1), which compares well with 1.50 × 10^-8^, a previously known rate in Arabideae [59]. However, this rate has since been revised to 7.5 × 10^-9 ^with the recent finding that the divergence time between *A. thaliana *and *A. lyrata *is about twice the previously known time, i.e. ~10 MYA instead of ~5 MYA [60].

**Figure 1:**
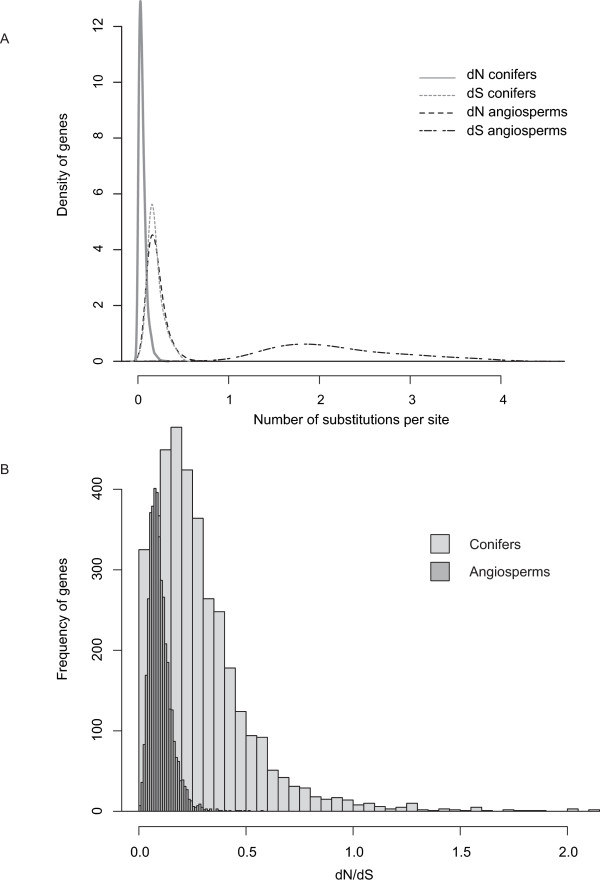
**Distribution of evolutionary estimates for conifer and angiosperm protein-coding genes**. A. Smoothed density plots of dS and dN estimates. B. Histogram plots of dN/dS estimates. Conifer genes with dN/dS > 2 (n = 38) are not shown. Breaks = 200.

We also found a difference in μ_N _between gymnosperms and angiosperms (0.18 × 10^-9 ^and 0.92 × 10^-9 ^mutations per year, respectively), representing a five-fold difference. If we account for the differential rate between the two angiosperm species, the difference for μ_N _is 9-fold and 1.5-fold with *Arabidopsis *and poplar, respectively (see Table 1). Figure 1.A illustrates the difference in dS and dN distributions between conifers and angiosperms, in particular the strikingly low dS estimates for conifers.

Overall, our results indicate a relative over-representation of non-synonymous mutations versus synonymous mutations in conifer species compared to angiosperm species. Consequently, mean dN/dS is higher in conifers than in angiosperms, i.e. 0.3137 and 0.0924, respectively, on average, and the distribution of dN/dS values for conifers extends towards and over unity (Figure 1.B). While we found 100 conifer genes with dN/dS > 1 out of 3,723 orthologues, there was a single *Arabidopsis*-poplar orthologue out of 4,080 orthologues that showed signs of positive selection over the entire alignment (dN/dS = 1.8565). This gene (*ORF25*; TAIR ID: ATMG00640; UniProt ID: Q04613) encodes a plant b subunit of mitochondrial ATP synthase.

**Figure 2:**
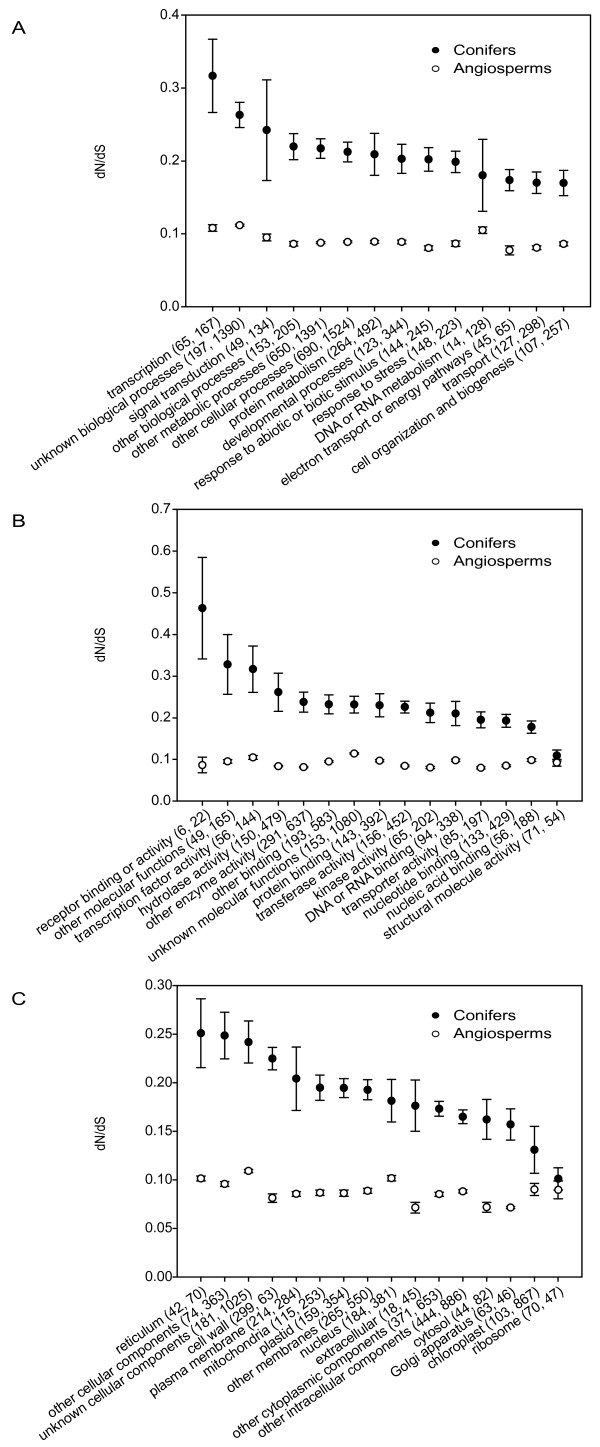
**dN/dS estimates in conifer and angiosperm genes across *Arabidopsis' *GO slim functional categories**. Mean dN/dS values for conifer (full circle) and angiosperm (open circle) protein-coding genes. Conifer genes were BLASTed against *Arabidopsis *gene transcripts, whose GO Slim annotations were used for homologous conifer genes. Brackets represent the standard error of the mean. A: Biological processes; B: Molecular functions; C: Cellular component.

We compared dN/dS between functional categories in conifers and gymnosperms, and consistently found higher dN/dS in conifers in most functional GO Slim categories (Figure 2; Mann-Whitney test, P < 0.05). However, 'DNA/RNA metabolism' (biological processes; P = 0.37), and 'chloroplast' and 'ribosome' (cellular component; P = 0.46 and P = 0.62, respectively) showed no significant difference.

If synonymous mutations, and even more so mutations at 4D sites, follow a neutral mode of evolution, we would expect no significant difference in average μ_S _between functional categories (Additional file 4). However, there were significant disparities among some of the functional categories, even when considering the 'more neutral' mutations at 4D sites (Kruskal-Wallis test; H = 52.831, P < 0.001), a surprising finding because it goes against the neutral expectancy. Interestingly, a recent study in birds has found evidence for selective constraints at 4D sites in the avian genome [61], and completes previous evidence accumulated in mammals [56]. Taken together, these results should call for careful attention when using dS as an estimate of neutral mutation rate, especially when inferring positive selection from dN/dS estimates or when applying molecular clocks. The present study does not claim positive selection but merely reports evolutionary trends; our results are therefore not significantly affected by the assumed neutrality of dS.

## Discussion

Our findings, based upon large-scale sampling rather than a small set of genes, are of significance for understanding the differences in patterns of evolution between conifers and angiosperms. First, we found that evolutionary rates are dramatically lower in conifers than in angiosperms. Second, we find that such differences vary across functional categories of genes.

Classically, interspecific studies of protein-coding genes in conifers have involved very few loci. Kusumi *et al. *[62] studied evolutionary rates of 11 genes in the Cupressacea. Bouillé and Bousquet [63] compared polymorphisms of three nuclear genes in *Picea*. More recently, Palmé *et al. *[64] scrutinized patterns of selection in 21 nuclear genes in a pine phylogeny while Chen *et al. *[65] carried out similar analyses for 10 genes in four spruce species. Large-scale comparative approaches are needed to grasp global evolutionary trends representative of conifer genomes.

Genome-scale sequencing of conifer genomes is coming of age [26,27], in particular for two economically and environmentally important species of the Pinaceae: Sitka spruce and loblolly pine. EST datasets for these species have previously been used in a comparative framework to find conifer-specific genes [66] and studying the evolution of gene families [67] and of xylem-specific genes [68] in vascular plants. Here, we carried out the first comparative study of substitution rates and mutational patterns in a sizable fraction of the conifer gene set - or that of any gymnosperm.

### Lower rates of evolution in conifers as compared to angiosperms

#### Are evolutionary rates slower in conifers and gymnosperms than in angiosperms?

We estimated evolutionary measures at 3,723 conifer orthologues and 4,080 angiosperm orthologues. As in any partial list of ESTs (i.e. not genome-wide), there might have been an unintentional selection of particular functional categories of genes, but we believe that our gene set is large enough to be representative of the genome as a whole. We found a much smaller dS in conifers than in angiosperms (0.1908 and 2.1846, respectively; see Table 1). A practical consequence of this difference is that we discarded almost 10 times as many angiosperm genes before final analysis; these genes showed a significant level of genetic saturation compared to conifer genes. Genetic saturation artificially reduces sequence divergence because multiple mutations at any given site of a particularly fast-evolving gene cannot be ruled out. All considered, not discarding these genes would only increase the difference in dS between conifers and angiosperms. Estimates of dN were also lower in conifers than in angiosperms (0.0492 and 0.2019, respectively), but the difference was not as dramatic as for dS (see Table 1, Figure 1.A), suggesting that substitutions at synonymous sites are particularly constrained - or that those at non-synonymous sites are less constrained, at equal mutation rate, in conifers as compared to angiosperms. Although the causes for this pattern of substitutions in conifer genes are unclear, the answer resides in what seems a unique picture of mutational processes and/or selective influences that affect conifer genes (see below).

Using published divergence times, we inferred an average synonymous mutation rate of 0.68 × 10^-9 ^substitutions per site per year in conifer genes (see Table 1); this is 15 times less than the average rate in 4,080 *Arabidopsis*-poplar orthologues (μ_S _= 9.93 × 10^-9^). If we account for the lower (1:6) rate in the poplar lineage [57], the difference is 25 times less in conifers than in *Arabidopsis *(μ_S _= 17.02 × 10^-9^), and four times less than poplar (μ_S _= 2.84 × 10^-9^). We compiled a list of substitution rates that have been published for gymnosperms and angiosperms (Additional File 5), and our findings fall well into the range of rates reported for the two seed plant groups. For example, two phytochrome genes were shown to evolve at a synonymous rate of 0.48 × 10^-9 ^per year in *Pinus sylvestris *and *Picea abies *[69]. For angiosperms, a rate of 1.5 × 10^-8 ^per year was commonly accepted for *Arabidopsis *[59] and the resulting 1:6 rate in poplar (2.5 × 10^-9 ^per year) is also very similar to our results (Table 1). However, with a divergence time between *A. thaliana *and *A. lyrata *recently revised at ~10 MY [60], the current estimate of the mutation rate in *Arabidopsis *has doubled. Although it is unclear how this relates to our results, it is important to acknowledge the uncertainty that exists in our results, in the 1:6 poplar:*Arabidopsis *ratio and in timing divergence, even when relaxed molecular clocks are used.

Interestingly, at the population level, conifers also exhibit lower nucleotide diversity despite high gene flow and low population structure [65,70,71]. In addition, low substitution rate and low nucleotide diversity in conifers are paralleled with reports of relatively low evolutionary rates above the nucleotide level. For example, angiosperms are highly diversified while gymnosperms have experienced a very low speciation rate [72]. At least in birds, diversification has been shown to be positively correlated with mutation rate [73]. At the chromosome level, not only is there little variation in the number of haploid conifer chromosomes (n = 11-13) with only scarce evidence of whole genome duplication and polyploidy [74] but comparative genome maps also suggest that macrosynteny is conserved; making it possible to easily navigate across genomes [75] and suggesting that conifer chromosomes are 'fossilized'. There is on the contrary, a high rate of chromosome evolution in angiosperms [72], as well as frequent polyploidy and genome duplication events. Finally, Jaramillo-Correa *et al. *[76] found that recombination, which has been correlated with levels of genetic diversity, is lower in conifers compared to angiosperms.

There are only a few known exceptions to this general trend of lower evolutionary rates in gymnosperms. Conifers have larger genomes than angiosperms [74], partly due to larger gene families and abundance of pseudogenes and partly due to a very high content in repetitive DNA such as transposable elements [27,74]. Possible elevated rates of gene duplication and transposition could have occurred along the gymnosperm lineage to cause this genome expansion, with evidence to date suggesting that these events were ancient [77]. Despite these exceptions, conifers exhibit dramatically slower evolutionary rates compared to angiosperms, in particular substitution rates in protein-coding genes, suggesting the existence of conifer-specific evolutionary mechanisms.

#### What are the causes for the slow substitution rates in conifer genomes?

Substitution rates vary depending on rates at which mutations appear in individuals and are fixed in the population [9,13].

First, the rate at which mutations appear is affected by the efficacy of the DNA repair machinery, generation time, and metabolic rate. In animals, mitochondrial genes evolve ten times faster than nuclear genes, but the inverse situation is found in plants [4]. This difference may at least in part originate from the presence of the DNA repair gene *recA *in plant mitochondrial genomes, and its absence in those of animals [78]. To our knowledge, there is no information on the efficiency of the conifer DNA repair system compared to that of angiosperm species. Life history traits such as generation time or total life span are factors that are commonly called forth to explain differences in evolutionary rates detected between species, e.g. in mammals [79], in invertebrates [80] and in plants [81]. In angiosperms, rates of evolution are higher in annuals than in perennials [15]. Our data supports this finding as *Arabidopsis *(an annual) has higher rates than trees. This accords with the germline theory of mutations [82]. However, generation time effects will be unknown until we can reconcile the difference between cell lineage division time and generation time in plants [14]. Conifers exhibit lower values of nucleotide diversity at the population level despite high gene flow and low population structure [65,70,71] suggesting that trees accumulate fewer mutations per unit of time than other plants and thus generation time is not sufficient to explain the annual-perennial difference in mutation rates. Finally, the low metabolite rate of conifer trees, with their large body size and temperate to boreal habitats [83], as well as reduced recombination rates [76], could generate fewer nucleotide substitutions in their genomes.

Second, the fixation rate of new mutations depends on the interplay between random genetic drift (i.e. effective population size and population structure), purifying (background) selection and reproductive strategy. Large population sizes and extensive gene flow are often suggested as the causes of low synonymous polymorphism found in conifer populations [58]. Both empirically and theoretically, grey areas remain about the effect of effective population size (N_e_), population subdivision and selection on the pattern of nucleotide divergence between species [84-86]. Our results however support the inverse relationship between N_e _and neutral substitution rate that is expected by the "nearly neutral theory of molecular evolution" [87]. In addition, with low diversification rate in conifers [72], there have been fewer speciation-associated bottleneck events than in angiosperms, thus continuous low diversity between populations. That conifers are mainly outcrossing (selfing is generally avoided through high early inbreeding depression) is only adding to the homogenization of populations. Indeed, studies have shown that there is weak population structure in Sitka spruce [88] and loblolly pine [89]. Finally, the influence of background selection and other selective forces such as hitchhiking on the genomic reduction of substitution rate in conifers is mostly unknown, although selective sweeps following bottlenecks have been reported for several loci [22,23,90].

Teasing out the evolutionary mechanisms controlling the rate of evolution in any organism is a daunting task. When comprehensive data are available across several conifer and other gymnosperm species, comparative analyses will help elucidate if, in what manner and to what extent typical conifer features such as low metabolite rate, long generation time, large effective population and low genetic structure affect substitution rates [91,92].

#### Is the evolutionary slow-down similar between conifer and angiosperm trees?

Conifers have high levels of genetic diversity within population but experience low nucleotide substitution rates and low speciation rates. Strikingly, the same trend can be seen in angiosperm trees and all trees (angiosperm and gymnosperm) share common attributes that may explain this similarity such as perenniality, outcrossed mating system and large population sizes [58,82]. However, vast evidences point at a more pronounced slow-down in conifers compared to angiosperm trees, for example: recombination rate [76], nucleotide diversity [58] and substitution rates. In this study, we found that conifers have a lower substitution rate at both synonymous and non-synonymous sites than poplar (see Table 1). The existence of conifer-specific factors that explain this difference is therefore likely; gymnosperms have evolved separately from angiosperms for about 300 MY. However, the exact nature and influence of these factors are still to be determined.

### High adaptability of conifers to their environment

We found that mean dN/dS was about three times higher in conifers than in angiosperms (0.3137 *vs*. 0.0924, respectively; see Table 1) despite much lower substitution rates in conifer protein-coding genes, and that this trend was found throughout almost all functional categories. Higher dN/dS in conifers could be due to a general low mutation rate and a high selective constraint on synonymous mutations, which seems at odds with the neutral expectancy but cannot be completely ruled out, or a general very low mutation rate but a proportionally lower constraint (relative to angiosperm genes) at non-synonymous sites. Assuming a relatively high rate of amino acid change in conifer proteins, high average estimates of dN/dS in conifers have important evolutionary implications, especially in light of the distinctive biology of conifer trees.

#### Characteristics of fast-evolving genes and functional gene categories

Among 100 conifer genes with dN/dS > 1, we found a large fraction of genes involved in abiotic and biotic stress response. For example, we found two protein phosphatases with dN/dS > 6, and one protein kinase with dN/dS~3 (see Table 2). Protein phosphatases and kinases act in tandem to regulate signaling pathways for plant stress tolerance or avoidance [93]. Four heat shock proteins, one leucine-rich repeat protein, one histone modification protein, two glycosyltransferases, four glycoside hydrolases, and seven transcription factors are also gene products involved in defense, resistance and/or stress response. Other genes with dN/dS > 1 were involved in cell signaling, development and growth, vesicle trafficking and DNA/RNA binding. These single-gene results were paralleled by a gene set analysis on 1,230 annotated genes ranked by dN/dS, where functional categories involved with heat shock proteins, signal transduction and in the regulation of transcription and nucleic acids were more likely to contain genes with high dN/dS (Additional File 3). Conifers, like other long-lived sessile plants, require responsiveness and plasticity to defend themselves against various herbivores and pathogens, as well as abiotic stresses (e.g. temperature and drought). This plasticity can for example be obtained by regulating transcription and DNA/RNA binding proteins, which could explain why these groups of genes seem to have experienced adaptive selection in conifer lineages. In contrast, categories of genes involved in translation, protein assembly, cellular organization and chlorophyll biosynthesis are under strong selective constraint (low dN/dS) because these processes are highly conserved across either the tree of Life, or across photosynthetic organisms (i.e. chlorophyll biosynthesis).

#### Adaptability of conifers

The conifer divergence was dramatically slower at synonymous sites than at non-synonymous sites (11-fold vs. 4-fold), suggesting that more adaptive mutations (and deleterious mutations, but see below) are fixed in conifers than in angiosperms. Indeed, there was a single *Arabidopsis*-poplar orthologue gene with a dN/dS > 1 while values for other orthologues were below 0.6. Conversely, we found a distribution of conifer dN/dS ratios significantly deviated near unity (Figure 1.B), with 100 genes showing values suggesting positive selection (dN/dS > 1). In addition, all GO Slim functional categories showed a significantly higher dN/dS in conifers than in angiosperms, with the exception of DNA/RNA metabolism and translation, which are evolutionary stable processes (Figure 2).

A threshold of unity is usually applied to determine if a gene shows signs of adaptive evolution, but this threshold is overly conservative in the case of pairwise comparisons over the whole length of the alignment. Algorithms exist to identify adaptive mutations at specific sites and/or on specific branches of a species tree, even when dN/dS < 1 over the entire gene, but there is an implicit requirement for comparisons of at least three species [3]. At the time of this study, loblolly pine and Sitka spruce had significantly more publicly available sequences than any other conifer, and we chose to restrain our study to two species and several thousands of genes, rather than opting for additional species but a few hundreds of genes. With more sequences becoming available for conifer species [94], it will be possible to test for positive selection using models of evolution across a tree composed of three or more species.

An overarching goal of modern biology is to uncover the genetic architecture of biological adaptations. Our study suggests that there is a substantial amount of adaptive substitutions in two conifer species and we expect that this finding will be generalized to other conifer taxa, especially in environments where conifers compete in extreme ecological niches. For example, the Vietnamese pine has evolved broad leaves, i.e. flattened needles, to compete for light with evergreen angiosperm trees in tropical forests [95]. In Western North America, lodgepole pine has evolved large and thick-scaled cones where squirrels are absent but crossbills are present, while crossbills evolve larger beaks [96]. An arms race between conifers and herbivorous insects, such as bark beetles, results in the diversification of constitutive defense and stress-induced genes in conifers [97]. Sitka spruce and loblolly pine, like most conifers in their natural environment, have been confronted by various endemic herbivorous pests, which we speculate could be reflected by high dN/dS estimates at genes involved in defense and stress response.

#### Why do conifers show more signs of adaptive evolution than most plant lineages?

Our results show that the low mutational rate seen in conifer genes is congruent with higher dN/dS, i.e. higher adaptability at the amino acid level, compared to angiosperm genes. At first, this relationship might seem contradictory and counter-intuitive; it is accepted that mutations are the foundation for adaptation. In conifers, a combination of factors seems to have promoted a staggering high rate of fixation for non-synonymous mutations, despite a generalized low mutation rate.

Little evidence has been found for adaptive evolution in angiosperm genes. In *Arabidopsis thaliana *and *A. lyrata*, purifying selection is the determinant force acting on amino acid substitutions [98]. In addition, Gossmann et al. [99] found little or no signal of adaptation in nine pairs of angiosperm species, except in sunflowers. Other exceptions to this rule are European aspen [100] and the crucifer *Capsella grandiflora *[101], where 30% and 40% of amino acid substitutions have been fixed by natural selection, respectively. What differentiates sunflowers and *C. grandiflora *from the other studied angiosperms are low population genetic structure and especially large effective population size (N_e _> 500,000). European aspen has a lower reported N_e _(118,000) but it has been argued that 500,000 individuals may not be unrealistic [100]. Strasburg et al. [102] compared different species of sunflowers, and found a positive correlation between N_e _and levels of adaptive divergence. Sunflowers, European aspen and *C. grandiflora *are also outcrossing species but an excess of non-synonymous mutations was found in the outcrossing *A. lyrata *[98], so mating system may only have limited effect on selective pressure compared to demographic factors. Lastly, selfing *A. thaliana *appears to have rare adaptive substitutions, likely due to consequent population subdivision and reduced N_e _through different bottleneck episodes [98,103,104].

In conifers, investigations of sequence divergence at the genome level have not been performed yet. Resequencing and comparative data have already provided a large body of evidence that several individual genes in conifers species have evolved under positive selection [58,64,89]. In addition, there are various examples of local adaptation in conifer species, whereby a specific population within the range of the species has expressed a phenotype adapted to an environmental constraint [105-107]. Concurrent with our results, the overall picture from the study of molecular evolution of conifer genes is that ecology, demography, life history and genome stability of conifers are favorable for the fixation of non-synonymous mutations. While fixation of deleterious mutations is reduced by outcrossing and large effective population size, most non-synonymous mutations are likely beneficial mutations in the conifer phyla. In addition, although deleterious mutations could be fixed through bottlenecks and selective sweeps, it has been shown that the time to establishment of complex adaptations is minimized in species with a large effective population size, even in the advent of deleterious intermediate steps [108].

## Conclusions

Large-scale and genomewide comparative approaches go beyond comparisons of small groups of candidate genes and provide global evolutionary trends. In this study, we found that there was a dramatic slow-down in the overall mutation rate of conifer orthologues compared to angiosperm orthologues. This finding is compatible with an increase in the fixation of non-synonymous mutations, which can be beneficial for adaptation. Large effective population size is likely the main factor that contributes to this trend, along with low population structure, low recombination and outcrossing mating system.

Several genome sequencing projects in conifer species are now funded including for loblolly pine, Douglas fir, sugar pine, white spruce and Norway spruce. These data will allow phylogenetic comparisons of much greater power then we currently employ. Not only should the present approach be expanded to a phylogenetic context, but future studies may also apply comparative methods to tease out the evolutionary processes under various demographic and ecological scenarios [91,92]. Finally, resequencing large numbers of candidate genes, once a reference genome sequence is established, will further identify the mode and strength of selection in conifer genomes.

## List of abbreviations

BAC: Bacterial Artificial Chromosome; cDNA: complementary DNA; EST: Expressed Sequence Tag; FLcDNA: Full-length cDNA; GO: Gene Ontology; MYA: Million Years Ago; ORF: Open Reading Frame; RBH: Reciprocal Best Hit; 4D: 4 fold degenerate.

## Authors' contributions

EB participated in the design of the study, performed the analyses, and drafted the manuscript. KR conceived of the study, and participated in its design, analysis, and final write-up. JB was involved with the initial grant proposal, with the identification of genes important for secondary metabolites, and grant leadership. CR was involved in project management. All authors read, revised and approved the final manuscript.

## Supplementary Material

Additional file 1 **Evolutionary measures for angiosperm and gymnosperm orthologues**. Includes gene/transcript/EST IDs, ORF length, aligned and analyzed length, and dN, dS and dN/dS estimates. Click here for file

Additional file 2 **Annotation and dN/dS values for conifer orthologous genes**. A more detailed description based on UniProt, PFAM and Interpro searches is provided for the 100 genes that showed dN/dS > 1, as well putative function where relevant. Click here for file

Additional file 3 **Gene set analyses of conifer annotated genes (Fatiscan and logistic regression methods)**. Includes references to Babelomics and statistical methods, and results of over-represented categories of genes with high and low dN/dS (adjuste p < 0.05, flase discovery rate correction) in InterPro, KEGG pathways, and GO functional cetegories. Click here for file

Additional file 4 **dS estimates in conifer and angiosperm genes across *Arabidopsis' *GO Slim functional categories**. Mean dS values for conifer (full circle) and angiosperm (open circle) protein-coding genes. Conifer genes were BLASTed against *Arabidopsis *gene transcripts, whose GO Slim annotations were used for homologous conifer genes. Brackets represent the standard error of the mean. A: Biological processes; B: Molecular functions; C: Cellular component. Click here for file

Additional file 5 **Literature survey for plant mutation rates**. Click here for file

## References

[B1] Nielsen R Molecular signatures of natural selection Annu Rev Genet 2005 39 197 218 10.1146/annurev.genet.39.073003.112420 16285858

[B2] Hurst LD Genetics and the understanding of selection Nat Rev Genet 2009 10 83 93 19119264 10.1038/nrg2506

[B3] Yang Z Nielsen R Codon-substitution models for detecting molecular adaptation at individual sites along specific lineages Mol Biol Evol 2002 19 908 917 10.1093/oxfordjournals.molbev.a004148 12032247

[B4] Wolfe KH Li WH Sharp PM Rates of nucleotide substitution vary greatly among plant mitochondrial, chloroplast, and nuclear DNAs Proc Natl Acad Sci USA 1987 84 9054 9058 10.1073/pnas.84.24.9054 3480529 PMC299690

[B5] Drouin G Daoud H Xia J Relative rates of synonymous substitutions in the mitochondrial, chloroplast and nuclear genomes of seed plants Mol Phylogenet Evol 2008 49 827 831 10.1016/j.ympev.2008.09.009 18838124

[B6] Britten RJ Rates of DNA sequence evolution differ between taxonomic groups Science 1986 231 1393 1398 10.1126/science.3082006 3082006

[B7] Kumar S Subramanian S Mutation rates in mammalian genomes Proc Natl Acad Sci USA 2002 99 803 808 10.1073/pnas.022629899 11792858 PMC117386

[B8] Nishant KT Singh ND Alani E Genomic mutation rates: what high-throughput methods can tell us Bioessays 2009 31 912 920 10.1002/bies.200900017 19644920 PMC2952423

[B9] Lanfear R Welch JJ Bromham L Watching the clock: Studying variation in rates of molecular evolution between species Trends Ecol Evol 2010 25 495 503 10.1016/j.tree.2010.06.007 20655615

[B10] Rocha EPC The quest for the universals of protein evolution Trends Genet 2006 22 412 416 10.1016/j.tig.2006.06.004 16808987

[B11] Pál C Papp B Lercher MJ An integrated view of protein evolution Nat Rev Genet 2006 7 337 348 10.1038/nrg1838 16619049

[B12] Warnecke T Weber CC Hurst LD Why there is more to protein evolution than protein function: splicing, nucleosomes and dual-coding sequence Biochem Soc Trans 2009 37 756 761 10.1042/BST0370756 19614589

[B13] Baer CF Miyamoto MM Denver DR Mutation rate variation in multicellular eukaryotes: causes and consequences Nat Rev Genet 2007 8 619 631 17637734 10.1038/nrg2158

[B14] Gaut B Yang L Takuno S Eguiarte LE The patterns and causes of variation in plant nucleotide substitution rates Annual Review of Ecology, Evolution, and Systematics 2011 42 245 266 10.1146/annurev-ecolsys-102710-145119

[B15] Yue JX Li J Wang D Araki H Tian D Yang S Genome-wide investigation reveals high evolutionary rates in annual model plants BMC Plant Biol 2010 10 242 10.1186/1471-2229-10-242 21062446 PMC3095324

[B16] Hedges SB Dudley J Kumar S TimeTree: a public knowledge-base of divergence times among organisms Bioinformatics 2006 22 2971 2972 10.1093/bioinformatics/btl505 17021158

[B17] Plomion C Chagné D Pot D Kumar S Wilcox P Burdon R Prat D Peterson D Paiva J Chaumeil P Kole C Pines Forest Trees 2007 Berlin Heidelberg: Springer-Verlag 29 92

[B18] Bousquet J Isabel N Pelgas B Cottrell J Rungis D Ritland K Kole C Spruce Forest Trees 2007 Berlin Heidelberg: Springer-Verlag 93 114

[B19] Hurme P Sillanpaa MJ Arjas E Repo T Savolainen O Genetic basis of climatic adaptation in Scots pine by Bayesian quantitative trait locus analysis Genetics 2000 156 1309 1322 11063704 10.1093/genetics/156.3.1309 PMC1461308

[B20] Ukrainetz N Ritland K Mansfield S Identification of quantitative trait loci for wood quality and growth across eight full-sib coastal Douglas-fir families Tree Genet Genom 2008 4 159 170 10.1007/s11295-007-0097-x

[B21] Pelgas B Bousquet J Meirmans P Ritland K Isabel N QTL mapping in white spruce: gene maps and genomic regions underlying adaptive traits across pedigrees, years and environments BMC Genomics 2011 12 145 10.1186/1471-2164-12-145 21392393 PMC3068112

[B22] Eckert AJ Wegrzyn JL Pande B Jermstad KD Lee JM Liechty JD Tearse BR Krutovsky KV Neale DB Multilocus patterns of nucleotide diversity and divergence reveal positive selection at candidate genes related to cold-hardiness in coastal Douglas-fir (*Pseudotsuga menziesii *var. *menziesii*) Genetics 2009 183 289 298 10.1534/genetics.109.103895 19596906 PMC2746152

[B23] Ersoz ES Wright MH González-Martínez SC Langley CH Neale DB Evolution of disease response genes in loblolly pine: insights from candidate genes PLoS ONE 2010 5 e14234 10.1371/journal.pone.0014234 21151911 PMC2997792

[B24] Quesada T Gopal V Cumbie WP Eckert AJ Wegrzyn JL Neale DB Goldfarb B Huber DA Casella G Davis JM Association mapping of quantitative disease resistance in a natural population of loblolly pine (*Pinus taeda *L.) Genetics 2010 186 677 686 10.1534/genetics.110.117549 20628037 PMC2954465

[B25] Holliday JA Ritland K Aitken SN Widespread, ecologically relevant genetic markers developed from association mapping of climate-related traits in Sitka spruce (*Picea sitchensis*) New Phytol 2010 188 501 514 10.1111/j.1469-8137.2010.03380.x 20663060

[B26] Hamberger B Hall D Yuen M Oddy C Hamberger B Keeling CI Ritland C Ritland K Bohlmann J Targeted isolation, sequence assembly and characterization of two white spruce (*Picea glauca*) BAC clones for terpenoid synthase and cytochrome P450 genes involved in conifer defence reveal insights into a conifer genome BMC Plant Biol 2009 9 106 10.1186/1471-2229-9-106 19656416 PMC2729077

[B27] Kovach A Wegrzyn J Parra G Holt C Bruening G Loopstra C Hartigan J Yandell M Langley C Korf I Neale D The *Pinus taeda *genome is characterized by diverse and highly diverged repetitive sequences BMC Genomics 2010 11 420 10.1186/1471-2164-11-420 20609256 PMC2996948

[B28] Pavy N Boyle B Nelson C Paule C Giguère I Caron S Parsons LS Dallaire N Bedon F Bérubé H Identification of conserved core xylem gene sets: conifer cDNA microarray development, transcript profiling and computational analyses New Phytol 2008 180 766 786 10.1111/j.1469-8137.2008.02615.x 18811621

[B29] Verne S Jaquish B White R Ritland C Ritland K Global transcriptome analysis of constitutive resistance to the white pine weevil in spruce Genome Biology and Evolution 2011 10.1093/gbe/evr069 PMC3296464 21852250

[B30] Keeling C Weisshaar S Ralph S Jancsik S Hamberger B Dullat H Bohlmann J Transcriptome mining, functional characterization, and phylogeny of a large terpene synthase gene family in spruce (Picea spp.) BMC Plant Biol 2011 11 43 10.1186/1471-2229-11-43 21385377 PMC3058080

[B31] Lippert DN Ralph SG Phillips M White R Smith D Hardie D Gershenzon J Ritland K Borchers CH Bohlmann J Quantitative iTRAQ proteome and comparative transcriptome analysis of elicitor-induced Norway spruce (*Picea abies*) cells reveals elements of calcium signaling in the early conifer defense response Proteomics 2009 9 10.1002/pmic.200800252 19105170

[B32] Hall DE Robert JA Keeling CI Domanski D Quesada AL Jancsik S Kuzyk MA Hamberger B Borchers CH Bohlmann J An integrated genomic, proteomic and biochemical analysis of (+)-3-carene biosynthesis in Sitka spruce (*Picea sitchensis*) genotypes that are resistant or susceptible to white pine weevil The Plant Journal 2011 65 936 948 10.1111/j.1365-313X.2010.04478.x 21323772

[B33] Ralph SG Chun HJ Kolosova N Cooper D Oddy C Ritland CE Kirkpatrick R Moore R Barber S Holt RA A conifer genomics resource of 200,000 spruce (*Picea *spp.) ESTs and 6,464 high-quality, sequence-finished full-length cDNAs for Sitka spruce (*Picea sitchensis*) BMC Genomics 2008 9 484 10.1186/1471-2164-9-484 18854048 PMC2579922

[B34] Lippert D Yuen M Bohlmann J Spruce proteome DB: a resource for conifer proteomics research Tree Genet Genom 2009 5 723 727 10.1007/s11295-009-0220-2

[B35] Treenomix - Conifer Forest Health http://www.treenomix.ca/

[B36] Schneider M Lane L Boutet E Lieberherr D Tognolli M Bougueleret L Bairoch A The UniProtKB/Swiss-Prot knowledgebase and its Plant Proteome Annotation Program J Proteomics 2009 72 567 573 10.1016/j.jprot.2008.11.010 19084081 PMC2689360

[B37] Hirsh AE Fraser HB Protein dispensability and rate of evolution Nature 2001 411 1046 1049 10.1038/35082561 11429604

[B38] Jordan IK Rogozin IB Wolf YI Koonin EV Essential genes are more evolutionarily conserved than are nonessential genes in bacteria Genome Res 2002 12 962 968 12045149 10.1101/gr.87702 PMC1383730

[B39] The Arabidopsis Information Resource http://www.arabidopsis.org/

[B40] JGI Genome Portal http://genomeportal.jgi-psf.org/

[B41] Smedley D Haider S Ballester B Holland R London D Thorisson G Kasprzyk A BioMart - biological queries made easy BMC Genomics 2009 10 22 10.1186/1471-2164-10-22 19144180 PMC2649164

[B42] Subramanian A Kaufmann M Morgenstern B DIALIGN-TX: greedy and progressive approaches for segment-based multiple sequence alignment Algorithms Mol Biol 2008 3 6 10.1186/1748-7188-3-6 18505568 PMC2430965

[B43] Yang Z PAML: a program package for phylogenetic analysis by maximum likelihood Comput Appl Biosci 1997 13 555 556 9367129 10.1093/bioinformatics/13.5.555

[B44] Yang Z PAML 4: Phylogenetic Analysis by Maximum Likelihood Mol Biol Evol 2007 24 1586 1591 10.1093/molbev/msm088 17483113

[B45] R Development Core Team R: A language and environment for statistical computing. R Foundation for Statistical Computing, Vienna, Austria. ISBN 3-900051-07-0, URL 2011 http://www.R-project.org/

[B46] Savard L Li P Strauss SH Chase MW Michaud M Bousquet J Chloroplast and nuclear gene sequences indicate late Pennsylvanian time for the last common ancestor of extant seed plants Proc Natl Acad Sci USA 1994 91 5163 5167 10.1073/pnas.91.11.5163 8197201 PMC43952

[B47] Wang XQ Tank DC Sang T Phylogeny and divergence times in Pinaceae: Evidence from three genomes Mol Biol Evol 2000 17 773 781 10779538 10.1093/oxfordjournals.molbev.a026356

[B48] Miller C Mesozoic conifers Bot Rev 1977 43 217 280 10.1007/BF02860718

[B49] Lin C-P Huang J-P Wu C-S Hsu C-Y Chaw S-M Comparative chloroplast genomics reveals the evolution of Pinaceae genera and subfamilies Genome Biol Evol 2010 2 504 517 10.1093/gbe/evq036 20651328 PMC2997556

[B50] Alvin KL Further conifers of the Pinaceae from the Wealden formation of Belgium 1960 Bruxelles: Institut Royal des Sciences Naturelles

[B51] Gernandt DS Magallón S Geada López G Zerón Flores O Willyard A Liston A Use of simultaneous analyses to guide fossil-based calibrations of Pinaceae phylogeny Int J Plant Sci 2008 169 1086 1099 10.1086/590472

[B52] Bell CD Soltis DE Soltis PS The age and diversification of the angiosperms re-revisited Am J Bot 2010 97 1296 1303 10.3732/ajb.0900346 21616882

[B53] Medina I Carbonell J Pulido L Madeira SC Goetz S Conesa A Tarraga J Pascual-Montano A Nogales-Cadenas R Santoyo J Babelomics: an integrative platform for the analysis of transcriptomics, proteomics and genomic data with advanced functional profiling Nucl Acids Res 2010 38 W210 213 10.1093/nar/gkq388 20478823 PMC2896184

[B54] Swarbreck D Wilks C Lamesch P Berardini TZ Garcia-Hernandez M Foerster H Li D Meyer T Muller R Ploetz L The Arabidopsis Information Resource (TAIR): gene structure and function annotation Nucleic Acids Res 2008 36 D1009 1014 17986450 10.1093/nar/gkm965 PMC2238962

[B55] Kimura M The Neutral Theory of Molecular Evolution 1983 Cambridge: Cambridge University Press

[B56] Chamary JV Parmley JL Hurst LD Hearing silence: non-neutral evolution at synonymous sites in mammals Nat Rev Genet 2006 7 98 108 10.1038/nrg1770 16418745

[B57] Tuskan GA Difazio S Jansson S Bohlmann J Grigoriev I Hellsten U Putnam N Ralph S Rombauts S Salamov A The genome of black cottonwood, *Populus trichocarpa *(Torr. & Gray) Science 2006 313 1596 1604 10.1126/science.1128691 16973872

[B58] Savolainen O Pyhäjärvi T Genomic diversity in forest trees Curr Opin Plant Biol 2007 10 162 167 10.1016/j.pbi.2007.01.011 17292660

[B59] Koch MA Haubold B Mitchell-Olds T Comparative evolutionary analysis of chalcone synthase and alcohol dehydrogenase loci in *Arabidopsis*, *Arabis*, and related genera (Brassicaceae) Mol Biol Evol 2000 17 1483 1498 11018155 10.1093/oxfordjournals.molbev.a026248

[B60] Beilstein MA Nagalingum NS Clements MD Manchester SR Mathews S Dated molecular phylogenies indicate a Miocene origin for Arabidopsis thaliana Proc Natl Acad Sci USA 2010 107 18724 18728 10.1073/pnas.0909766107 20921408 PMC2973009

[B61] Künstner A Nabholz B Ellegren H Significant selective constraint at 4-fold degenerate sites in the avian genome and its consequence for detection of positive selection Gen Biol Evol 2011 10.1093/gbe/evr112 PMC3242499 22042333

[B62] Kusumi J Tsumura Y Yoshimaru H Tachida H Molecular evolution of nuclear genes in Cupressacea, a group of conifer trees Mol Biol Evol 2002 19 736 747 10.1093/oxfordjournals.molbev.a004132 11961107

[B63] Bouillé M Bousquet J Trans-species shared polymorphisms at orthologous nuclear gene loci among distant species in the conifer *Picea *(Pinaceae): implications for the long-term maintenance of genetic diversity in trees Am J Bot 2005 92 63 73 10.3732/ajb.92.1.63 21652385

[B64] Palmé A Pyhäjärvi T Wachowiak W Savolainen O Selection on nuclear genes in a *Pinus *phylogeny Mol Biol Evol 2009 26 893 905 10.1093/molbev/msp010 19168564

[B65] Chen J Kallman T Gyllenstrand N Lascoux M New insights on the speciation history and nucleotide diversity of three boreal spruce species and a Tertiary relict Heredity 2010 104 3 14 10.1038/hdy.2009.88 19639012

[B66] Ujino-Ihara T Tsumura Y Screening for genes specific to coniferous species Tree Physiology 2008 28 1325 1330 10.1093/treephys/28.9.1325 18595844

[B67] Volokita M Rosilio-Brami T Rivkin N Zik M Combining comparative sequence and genomic data to ascertain phylogenetic relationships and explore the evolution of the large GDSL-lipase family in land-plants Mol Biol Evol 2010 10.1093/molbev/msq226 20801908

[B68] Li X Wu H Southerton S Comparative genomics reveals conservative evolution of the xylem transcriptome in vascular plants BMC Evol Biol 2010 10 190 10.1186/1471-2148-10-190 20565927 PMC2907377

[B69] García-Gil MR Mikkonen M Savolainen O Nucleotide diversity at two phytochrome loci along a latitudinal cline in *Pinus sylvestris* Mol Ecol 2003 12 1195 1206 10.1046/j.1365-294X.2003.01826.x 12694283

[B70] Heuertz M De Paoli E Kallman T Larsson H Jurman I Morgante M Lascoux M Gyllenstrand N Multilocus patterns of nucleotide diversity, linkage disequilibrium and demographic history of Norway spruce [*Picea abies *(L.) Karst] Genetics 2006 174 2095 2105 10.1534/genetics.106.065102 17057229 PMC1698656

[B71] Pyhäjärvi T Garcia-Gil MR Knurr T Mikkonen M Wachowiak W Savolainen O Demographic history has influenced nucleotide diversity in European *Pinus sylvestris *populations Genetics 2007 177 1713 1724 10.1534/genetics.107.077099 18039881 PMC2147978

[B72] Levin DA Wilson AC Rates of evolution in seed plants: Net increase in diversity of chromosome numbers and species numbers through time Proc Natl Acad Sci USA 1976 73 2086 2090 10.1073/pnas.73.6.2086 16592327 PMC430454

[B73] Lanfear R Ho SYW Love D Bromham L Mutation rate is linked to diversification in birds Proc Natl Acad Sci USA 2010 107 20423 20428 10.1073/pnas.1007888107 21059910 PMC2996675

[B74] Ahuja MR Neale DB Evolution of genome size in conifers Silvae Genet 2005 54 126 137

[B75] Pelgas B Beauseigle S Achere V Jeandroz S Bousquet J Isabel N Comparative genome mapping among *Picea glauca, P. mariana *× *P. rubens *and *P. abies*, and correspondence with other Pinaceae Theor Appl Genet 2006 113 1371 1393 10.1007/s00122-006-0354-7 17061103

[B76] Jaramillo-Correa J Verdu M Gonzalez-Martinez S The contribution of recombination to heterozygosity differs among plant evolutionary lineages and life-forms BMC Evol Biol 2010 10 22 10.1186/1471-2148-10-22 20100325 PMC2826329

[B77] Friesen N Brandes A Heslop-Harrison JS Diversity, origin, and distribution of retrotransposons (*gypsy *and *copia*) in conifers Mol Biol Evol 2001 18 1176 1188 10.1093/oxfordjournals.molbev.a003905 11420359

[B78] Lin Z Kong H Nei M Ma H Origins and evolution of the *recA*/*RAD51 *gene family: Evidence for ancient gene duplication and endosymbiotic gene transfer Proc Natl Acad Sci USA 2006 103 10328 10333 10.1073/pnas.0604232103 16798872 PMC1502457

[B79] Welch J Bininda-Emonds O Bromham L Correlates of substitution rate variation in mammalian protein-coding sequences BMC Evol Biol 2008 8 53 10.1186/1471-2148-8-53 18284663 PMC2289806

[B80] Thomas JA Welch JJ Lanfear R Bromham L A generation time effect on the rate of molecular evolution in invertebrates Mol Biol Evol 2010 27 1173 1180 10.1093/molbev/msq009 20083649

[B81] Smith SA Donoghue MJ Rates of molecular evolution are linked to life history in flowering plants Science 2008 322 86 89 10.1126/science.1163197 18832643

[B82] Petit RJ Hampe A Some evolutionary consequences of being a tree Annu Rev Ecol Evol Syst 2006 37 187 214 10.1146/annurev.ecolsys.37.091305.110215

[B83] Gillooly JF Allen AP West GB Brown JH The rate of DNA evolution: Effects of body size and temperature on the molecular clock Proc Natl Acad Sci USA 2005 102 140 145 10.1073/pnas.0407735101 15618408 PMC544068

[B84] Whitlock MC Fixation probability and time in subdivided populations Genetics 2003 164 767 779 12807795 10.1093/genetics/164.2.767 PMC1462574

[B85] Woolfit M Effective population size and the rate and pattern of nucleotide substitutions Biol Lett 2009 5 417 420 10.1098/rsbl.2009.0155 19364708 PMC2679941

[B86] Li J Li H Jakobsson M Li SEN SjÖDin PER Lascoux M Joint analysis of demography and selection in population genetics: where do we stand and where could we go? Mol Ecol 2011 10.1111/j.1365-294X.2011.05308.x 21999307

[B87] Ohta T The nearly neutral theory of molecular evolution Annu Rev Ecol Syst 1992 23 263 286 10.1146/annurev.es.23.110192.001403

[B88] Gapare WJ Aitken SN Strong spatial genetic structure in peripheral but not core populations of Sitka spruce [*Picea sitchensis *(Bong.) Carr.] Mol Ecol 2005 14 2659 2667 10.1111/j.1365-294X.2005.02633.x 16029468

[B89] Eckert AJ van Heervaarden J Wegrzyn JL Nelson CD Ross-Ibarra J Gonzalez-Martinez SC Neale DB Patterns of population structure and environmental associations to aridity across the range of Loblolly pine (*Pinus taeda *L., Pinaceae) Genetics 2010 185 969 982 10.1534/genetics.110.115543 20439779 PMC2907212

[B90] Palmé AE Wright M Savolainen O Patterns of divergence among conifer ESTs and polymorphism in *Pinus sylvestris *identify putative selective sweeps Mol Biol Evol 2008 25 2567 2577 10.1093/molbev/msn194 18775901

[B91] Mayrose I Otto SP A likelihood method for detecting trait-dependent shifts in the rate of molecular evolution Mol Biol Evol 2011 28 759 770 10.1093/molbev/msq263 20961959

[B92] Lanfear R The local-clock permutation test: a simple test to compare rates of molecular evolution on phylogenetic trees Evolution 2011 65 606 611 10.1111/j.1558-5646.2010.01160.x 21272000

[B93] Chae L Pandey GK Luan S Cheong YH Kim KN Pareek A, Sopory SK Protein kinases and phosphatases for stress signal transduction in plants Abiotic Stress Adaptation in Plants 2010 Bohnert HJ: Springer Netherlands 123 163

[B94] Rigault P Boyle B Lepage P Cooke JEK Bousquet J MacKay JJ A white spruce gene catalog for conifer genome analyses Plant Physiol 2011 157 14 28 10.1104/pp.111.179663 21730200 PMC3165865

[B95] Brodribb TJ Feild TS Evolutionary significance of a flat-leaved *Pinus *in Vietnamese rainforest New Phytol 2008 178 201 209 10.1111/j.1469-8137.2007.02338.x 18179604

[B96] Benkman C Diversifying coevolution between crossbills and conifers Evo Edu Outreach 2010 3 47 53 10.1007/s12052-009-0190-8

[B97] Raffa KF Berryman AA Interacting selective pressures in conifer-bark beetle systems: A basis for reciprocal adaptations? Amer Nat 1987 129 234 262 10.1086/284633

[B98] Foxe JP Dar VU Zheng H Nordborg M Gaut BS Wright SI Selection on amino acid substitutions in *Arabidopsis* Mol Biol Evol 2008 25 1375 1383 10.1093/molbev/msn079 18390851 PMC2878001

[B99] Gossmann TI Song B-H Windsor AJ Mitchell-Olds T Dixon CJ Kapralov MV Filatov DA Eyre-Walker A Genome wide analyses reveal little evidence for adaptive evolution in many plant species Mol Biol Evol 2010 27 1822 1832 10.1093/molbev/msq079 20299543 PMC2915642

[B100] Ingvarsson PK Natural selection on synonymous and nonsynonymous mutations shapes patterns of polymorphism in *Populus tremula* Mol Biol Evol 2010 27 650 660 10.1093/molbev/msp255 19837657

[B101] Slotte T Foxe JP Hazzouri KM Wright SI Genome-wide evidence for efficient positive and purifying selection in *Capsella grandiflora*, a plant species with a large effective population size Mol Biol Evol 2010 27 1813 1821 10.1093/molbev/msq062 20194429

[B102] Strasburg JL Kane NC Raduski AR Bonin A Michelmore R Rieseberg LH Effective population size is positively correlated with levels of adaptive divergence among annual sunflowers Mol Biol Evol 2011 28 1569 1580 10.1093/molbev/msq270 20952500 PMC3080132

[B103] Nordborg M Hu TT Ishino Y Jhaveri J Toomajian C Zheng H Bakker E Calabrese P Gladstone J Goyal R The pattern of polymorphism in *Arabidopsis thaliana* PLoS Biol 2005 3 e196 10.1371/journal.pbio.0030196 15907155 PMC1135296

[B104] Cao J Schneeberger K Ossowski S Gunther T Bender S Fitz J Koenig D Lanz C Stegle O Lippert C Whole-genome sequencing of multiple *Arabidopsis thaliana *populations Nat Genet 2011 43 956 963 10.1038/ng.911 21874002

[B105] King JN Alfaro RI Cartwright C Genetic resistance of Sitka spruce (*Picea sitchensis*) populations to the white pine weevil (*Pissodes strobi*): distribution of resistance Forestry 2004 77 269 278 10.1093/forestry/77.4.269

[B106] Mimura M Aitken SN Local adaptation at the range peripheries of Sitka spruce J Evol Biol 2010 23 249 258 10.1111/j.1420-9101.2009.01910.x 20021549

[B107] Grivet D Sebastiani F Alia R Bataillon T Torre S Zabal-Aguirre M Vendramin GG Gonzalez-Martinez SC Molecular footprints of local adaptation in two Mediterranean conifers Mol Biol Evol 2010 28 101 116 20656795 10.1093/molbev/msq190

[B108] Lynch M Abegg A The rate of establishment of complex adaptations Mol Biol Evol 2010 27 1404 1414 10.1093/molbev/msq020 20118190 PMC3299285

