# Supplementary material for: Slow but not low: genomic comparisons reveal slower evolutionary rate and higher dN/dS in conifers compared to angiosperms
Source: BMC Evol Biol. 2012 Jan 20;12:8. doi: 10.1186/1471-2148-12-8 (PMC3328258; doi:10.1186/1471-2148-12-8)
Supplement: Additional file 4 — dS estimates in conifer and angiosperm genes across Arabidopsis' GO Slim functional categories. Mean dS values for conifer (full circle) and angiosperm (open circle) protein-coding genes. Conifer genes were BLASTed against Arabidopsis gene transcripts, whose GO Slim annotations were used for homologous conifer genes. Brackets represent the standard error of the mean. A: Biological processes; B: Molecular functions; C: Cellular component. [file 1471-2148-12-8-S4.PDF]

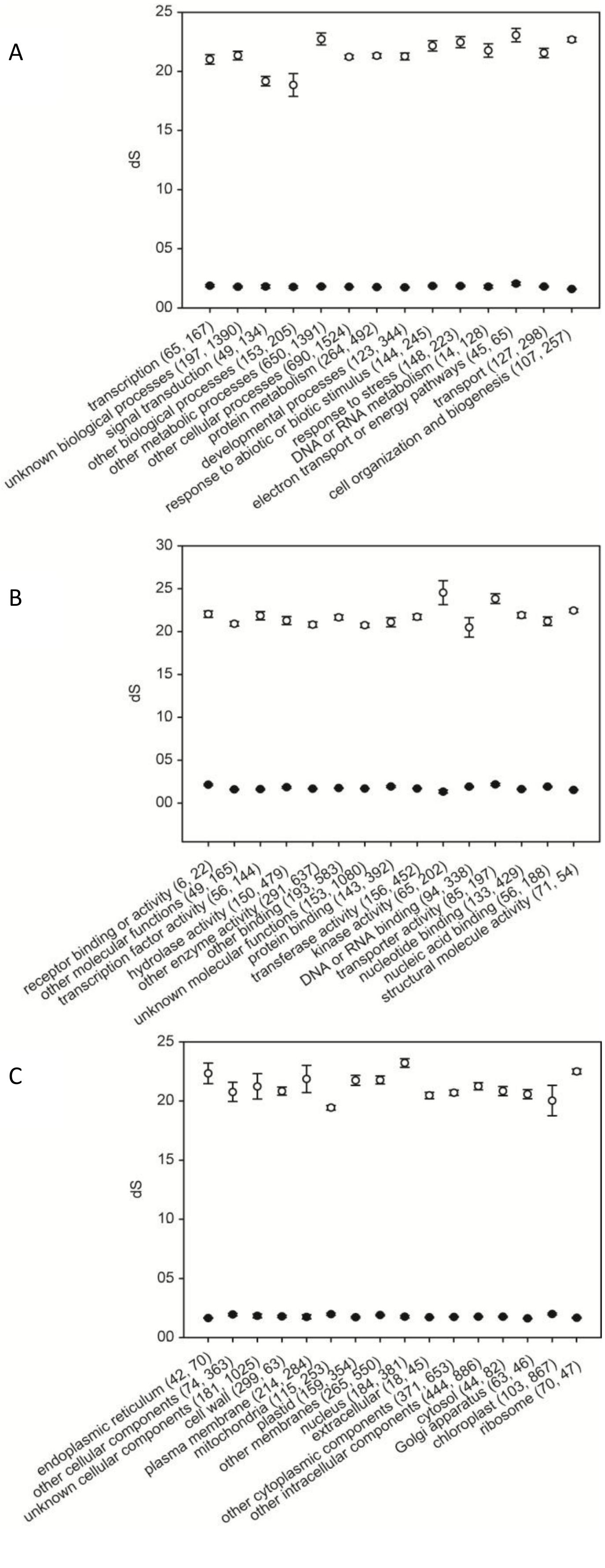
A
25
20
15
10
05
00
dS
transcription (65, 167)
unknown biological processes (197, 1390)
signal transduction (49, 134)
other biological processes (153, 205)
other metabolic processes (650, 1391)
other cellular processes (690, 1524)
protein metabolism (264, 492)
developmental processes (123, 344)
response to abiotic or biotic stimulus (144, 245)
response to stress (148, 223)
DNA or RNA metabolism (14, 128)
electron transport or energy pathways (45, 65)
transport (127, 298)
cell organization and biogenesis (107, 257)
B
30
25
20
15
10
05
00
dS
receptor binding or activity (6, 22)
other molecular functions (49, 165)
transcription factor activity (56, 144)
hydrolase activity (150, 479)
other enzyme activity (291, 637)
other binding (193, 583)
unknown molecular functions (153, 1080)
protein binding (143, 392)
transferase activity (156, 452)
kinase activity (65, 202)
DNA or RNA binding (94, 338)
transporter activity (85, 197)
nucleotide binding (133, 429)
nucleic acid binding (56, 188)
structural molecule activity (71, 54)
C
25
20
15
10
05
00
dS
endoplasmic reticulum (42, 70)
other cellular components (74, 363)
unknown cellular components (181, 1025)
cell wall (299, 63)
plasma membrane (214, 284)
mitochondria (115, 253)
plastid (159, 354)
other membranes (265, 550)
nucleus (184, 381)
extracellular (18, 45)
other cytoplasmic components (371, 653)
other intracellular components (444, 886)
cytosol (44, 82)
Golgi apparatus (63, 46)
chloroplast (103, 867)
ribosome (70, 47)

Additional file 4 – dS estimates in conifer and angiosperm genes across *Arabidopsis'* GO Slim functional categories.

Mean dS values for conifer (full circle) and angiosperm (open circle) protein-coding genes. Conifer genes were BLASTed against *Arabidopsis* gene transcripts, whose GO Slim annotations were used for homologous conifer genes. Brackets represent the standard error of the mean. A: Biological processes; B: Molecular functions; C: Cellular component.
